# Biomechanical effects of different bone conditions on oblique lumbar interbody fusion augmented with various fixation options: a finite element analysis

**DOI:** 10.3389/fbioe.2026.1815484

**Published:** 2026-08-17

**Authors:** Zhiqiang Wang, Yue yue Shi, Xin Peng, Tong Zhang, Hongqiang Wang, Guang Yang, Yanzheng Gao, Jia Shao

**Affiliations:** 1 Henan Provincial People’s Hospital, Zhengzhou, China; 2 Henan Cancer Hospital, Zhengzhou, China

**Keywords:** biomechanics, finite element analysis, oblique lumbar interbody fusion, osteoporosis, unilateral

## Abstract

**Objective:**

The purpose of this study was to analyze the biomechanical properties of oblique lumbar interbody fusion (OLIF) combined with various fixation methods under different bone conditions (normal bone, osteopenia and osteoporosis).

**Methods:**

The L3-L5 finite element (FE) models of normal bone (NB), osteopenia (OS) and osteoporosis (OP) were constructed. The OLIF combined with four kinds of internal fixation surgery models were constructed: OLIF Stand-alone (SA) ; OLIF + lateral plate fixation (OLIF + LP) ; OLIF + unilateral pedicle screw fixation (OLIF + UPS) ; OLIF + bilateral pedicle screw fixation (OLIF + BPS). A load of 500 N and a torque of 7.5 Nm were applied to the upper surface of L3 to simulate flexion (FL), extension (EX), left bending (LB), right bending (RB), left rotation (LR) and right rotation (RR). The range of motion (ROM) of the model, the maximum stress of Cage, L5 upper endplate and internal fixation were recorded.

**Results:**

(1) ROM comparison: Compared with the intact model, SA ROM decreased the least, BPS decreased the most, LP decreased significantly in the LB and RB, and was less stable in the FL and EX. (2) Stress comparison: the stress of SA endplate is the largest, the stress of BPS is the smallest, and the stress of LP is lower than that of UPS in LB and RB. The Cage stress is lower than its yield strength. The fixation stress of LP was roughly the same as that of BPS in LB and RB. In the NB and OS models, the fixation stress of LP under FL exceeds the minimum fatigue strength of the material by 22.72% and 33.78%, respectively. In the OP, the fixation stress exceeds the minimum fatigue strength of the material at FL and EX, and reaches 53.25% (>50%) at FL.

**Conclusion:**

OLIF supplemented with internal fixation can improve segment stability, and BPS is superior to UPS and LP fixation in limiting segment ROM and reducing stress. Under OP, the LP fixation stress at FL and EX exceeds the minimum fatigue strength of the material, which may increase the risk of fracture and internal fixation failure.

## Introduction

OLIF has been widely used in the treatment of lumbar degenerative diseases (Abe et al., 2017; Woods et al., 2017). But the choice of internal fixation remains controversial. Posterior BPS fixation is considered to be the gold standard for auxiliary fixation (Ambati et al., 2015). It has the advantages of good stability and high fusion rate (Song et al., 2021; Woods et al., 2017). However, posterior BPS fixation requires additional posterior incision, and needs to destroy part of the posterior column structure, resulting in large trauma and bleeding (Wen et al., 2020). UPS fixation further reduces surgical trauma (Wen et al., 2020; Chen et al., 2015), but posterior fixation inevitably requires intraoperative changes in position. The process of changing from lateral to prone position undoubtedly increases the risk of fusion cage displacement and anesthesia (Amato et al., 2010; Lau et al., 2013). With the development of minimally invasive technology, lateral screw (LSR) fixation (Xie et al., 2020; Guo et al., 2020) and lateral plate (LP) fixation (Cappuccino et al., 2010) have been applied to the auxiliary fixation of OLIF, and satisfactory clinical results have been achieved. They can be completed under the same incision, avoiding intraoperative position changes and reducing surgical trauma and time. In contrast, the lateral locking plate system is more round and blunt than the lateral screw, reducing the grinding effect on the surrounding tissue (Kaibara et al., 2010), and has been shown to provide fixed stability (Song et al., 2021; Zhang et al., 2018). However, the results of the above lateral fixation studies are based on normal bone mass, and lumbar degenerative diseases mostly occur in the elderly with different degrees of bone loss. Under OS and OP, whether LP fixation can effectively maintain intervertebral stability, and whether the presence of osteoporosis will affect the choice of internal fixation during surgery, there is still a lack of relevant research. Therefore, in this study, the FE method was used to compare and analyze the effects of internal fixation type and osteoporosis on the biomechanical properties of lumbar spine, aiming to provide a certain mechanical basis for clinical application.

## Materials and methods

### Construction of an intact lumbar finite element model

In Mimics20.0 (Materalise, Inc., Belgium), a complete lumbar L3-L5 FE model was constructed using a CT of a 35-year-old healthy male who excluded any spinal abnormalities. This study was approved by the ethics committee (Approval No:V3A02024124011). Combined with 3-Matic (Materalise, Inc., Belgium) software, the model was filled, smoothed and wrapped to obtain a preliminary lumbar 3D model. The model was optimized by reverse engineering software Geomagic Wrap 2017 (Geomagic, USA). The material properties of cortical bone, cancellous bone and endplate are defined as isotropic, homogeneous and linear elastic materials (Lu and Lu, 2019). The cartilage endplate is closely connected to the upper and lower parts of the vertebral body, with a thickness of about 1 mm. The cancellous bone is closely attached to the cortical bone. The thickness of the articular cartilage is about 2 mm, which is closely attached to the articular surface of the articular process. The nucleus pulposus and annulus fibrosus were simulated. The nucleus pulposus accounted for 44% of the entire intervertebral disc area, and the position was 3.5 mm behind the center of the intervertebral disc. The remaining structure was the annulus fibrosus (Liu et al., 2017; Zhao et al., 2018). Taking into account the computational efficiency of the model and the affordability of the model scale. The seven ligaments connecting the vertebrae were modeled as three-dimensional springs with only tension (Du et al., 2016).

### OLIF surgical models

According to the parameters of the solid model, each part is reversely generated in SolidWorks 2018 software. The Cage is 50 mm long, 18 mm wide, 12 mm high, 8°sagittal angle, inverted tooth polyhedral structure to increase the stability of the fixation. The pedicle screw was 6.5 mm in diameter and 45.0 mm in length, and the connecting rod was 5.5 mm in diameter and 53.0 mm in length. The LP device included a pair of cortical bone screws (nut diameter 7.5 mm; screw length 50 mm) and side plate (length 45 mm, the narrowest part of the middle 4 mm, screw hole diameter 10 mm, screw hole spacing 10 mm), screw and plate horizontal center line 15°angle. The bottom of the side plate is designed by light arc. The L4-L5 segment was defined as the surgical segment. Based on the NB, OS and OP L3-5 models, the OLIF Standalone (SA) model, the lateral plate fixation model (LP), the unilateral pedicle screw fixation model (UPS) and the bilateral pedicle screw fixation model (BPS) were constructed respectively. NB: SA model (M0), LP model (M1), UPS model (M2), BPS model (M3); OS: SA model (N0), LP model (N1), UPS model (N2), BPS model (N3); OP: SA model (P0), LP model (P1), UPS model (P2), BPS model (P3).

**FIGURE 1:**
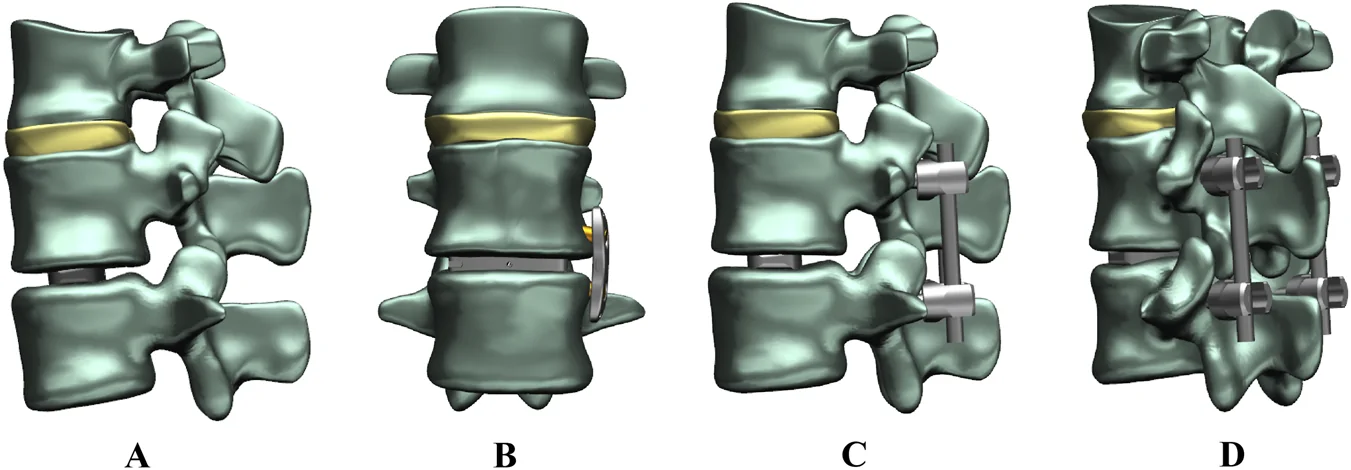
OLIF Surgical model. **(A)**: OLIF Standalone (SA); **(B)**: The lateral plate fixation model (LP); **(C)**: The unilateral pedicle screw fixation model (UPS); **(D)**: The bilateral pedicle screw fixation model (BPS).

The annulus fibrosus, nucleus pulposus and cartilage endplate were isolated and obliquely inserted into the Cage to simulate the SA model. On the basis of SA, the lateral plate was installed on the upper and lower edges of the lateral side of the L4-L5 vertebral body, and two cortical bone screws were placed across the bilateral epiphysis through the lateral plate to complete the fixation and simulate the LP model. On the basis of SA, two pedicle screws and one rod were implanted unilaterally to simulate UPS fixation, and four pedicle screws and two rods were assembled bilaterally to simulate BPS model Figures 1A–D.

### Diferent bone density models

According to previous studies (Polikeit et al., 2003), osteoporosis changes the elastic modulus of all bones in the vertebral body, while other material parameters do not change significantly. When the FE model of osteoporosis was established, the elastic modulus of all bone structures decreased, cancellous bone decreased by 66%, cortical bone, endplate and posterior structure decreased by 33%, and other structures remained unchanged (Salvatore et al., 2018; Su et al., 2017). Since osteopenia is a continuous range of T values, its model cannot simulate continuous variables. Therefore, the average elastic modulus of normal bone and osteoporosis is selected to represent osteopenia. The material properties and element types of the three bone components from studies by Chen et al. (2020), Shen et al. (2021), and Kumaran et al. (2021) (Table 1).

**TABLE 1:** Material properties of finite element models.

Component	Young’s modulus (MPa)	Poisson’s ratio	Stiffness
Cortical bone (NB)	12,000	0.3	—
Cortical bone (OS)	10,020	0.3	—
Cortical bone (OP)	8,040	0.3	—
Cancellous bone (NB)	100	0.2	—
Cancellous bone (OS)	67	0.2	——
Cancellous bone (OP)	34	0.2	—
Endplate	2000	0.2	—
Articular cartilage	25	0.25	—
Nucleus pulposus	1	0.50	—
Annulus	4.2	0.45	—
Cage peek	3,600	0.25	—
Pedicle screws and rod	110,000	0.28	—
Anterior longitudinal ligament	7.8	—	8.74
Posterior longitudinal ligament	10	—	5.83
Supraspinous ligament	8	—	15.38
Interspinous ligament	10	—	0.19
Ligamentum flavum	15	—	15.75
Transverse ligament	10	—	2.39
Capsular ligament	7.5	—	1.80

### Material properties, boundary, and loading conditions

The contact type between the models is defined in the connection. In this study, the constructed Cage is connected to the upper and lower end plates using a non-separated contact point. The contact surface of the fuse and the contact surface of the end plate are tooth-shaped anti-dislocation structures, and the friction coefficient is 0.8. The articular cartilage contact type (Rohlmann et al., 2006) was set to “frictional”, the coefficient was set to 0.2, the facet joint contact type was “no separation”, and the contact types such as screw and screw path, screw and connecting rod were set to “bonded” mode to simulate the immediate postoperative state (Ayturk et al., 2010). The lower surface of the L5 vertebral body was fixed. During the whole loading process, all the degrees of freedom of the lower endplate of the L5 vertebral body did not shift and rotate under the condition of force. A vertical load of 500 N was added to the upper surface of L3 to simulate the physiological load in the upright state, and a pure torque of 7.5 N·m was applied to simulate the movement in six directions: bending (FL), stretching (EX) ; left bending (LB), right bending (RB), left rotation (LR), right rotation (RR).

### Mesh sensitivity check

The established lumbar spine model was verified by patch sensitivity test. Axial rotation (AR) is considered to be the most sensitive to grid convergence (Ayturk and Puttlitz, 2011), and von mises stress is used as a criterion to judge the convergence of the grid. The mesh resolution is 1 mm, and the element sizes are 1 mm, 1.5 mm and 2 mm, respectively. When the difference between the prediction results of the two grid resolutions is less than 5%, the grid is considered to be convergent (Jones and Wilcox, 2008). The bottom of L5 was fixed, and a pure torque of 7.5 Nm was applied to the upper surface of L3. The results show that Mesh1, Mesh 2 and Mesh 3 contain 1,020 and 925 respectively. 865, 679 and 556, 498 elements. The difference between most stresses in mesh 1, mesh 2 and mesh 3 is less than 5% (Table 2). Therefore, it is proved that the mesh of the lumbar spine model is convergent and the patch repair is appropriate.

**TABLE 2:** Mesh sensitivity analysis.

Maximum mises stresses	Mesh1	Mesh2 (vs. Mesh1, difference)	Mesh3 (vs. Mesh2, difference)
Cortical bone (MPa)	5.62	5.54 (1.4%)	5.51 (2.0%)
Cancellous bone (MPa)	2.25	2.23 (1.0%)	2.19 (2.7%)
Nucleus (KPa)	9.78	9.36 (4.3%)	9.45 (3.4%)
Facet (KPa)	3.86	3.71 (3.9%)	3.69 (4.4%)

## Results

### Model validation

**FIGURE 2:**
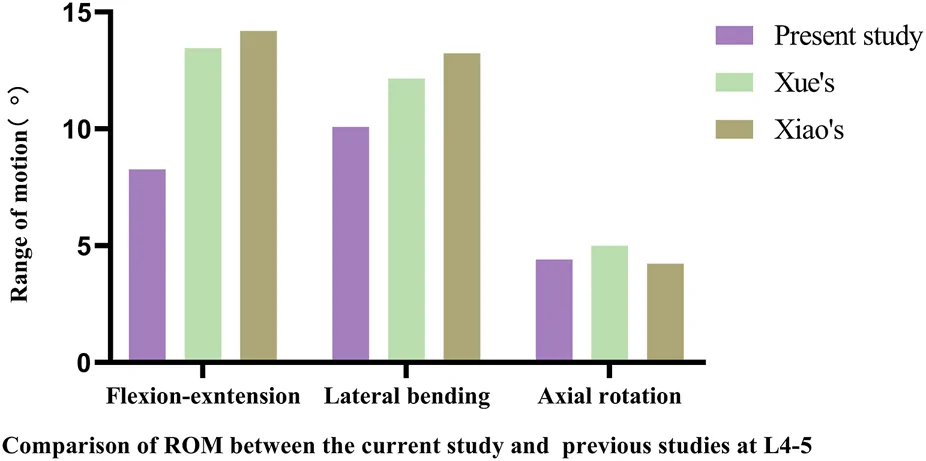
Comparison of ROM between the current study and previous studies at L4-5.

We compared the range of motion (ROM) results of this study with the results of Xue and Wu (2023), and Xiao et al. (2012), and found that the results of each model in all directions (flexion and extension, bending, rotation) were consistent with the results reported in the literature. The validity of the FE model is verified (Figure 2).

### ROM of surgical segments

**FIGURE 3:**
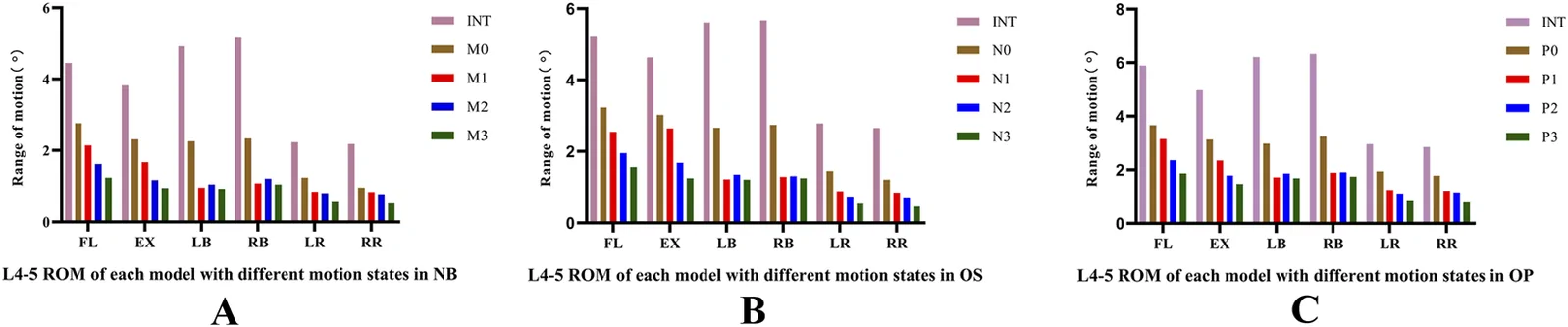
Comparison of L4-5 ROM under different bones and different motion states of each FE model. **(A)** NB; **(B)** OS; **(C)** OP.

Compared with the complete model, the stability of each surgical model increased, and the increase of BPS was the most obvious. Among the surgical models, the ROM of the M0 model decreased the least, and SA could not effectively reduce the flexion and extension and axial rotation. Compared with M0, M1 ROM decreases most obviously in the LB and RB directions, but has weak stability in the control FE direction. Compared with M1, M2 ROM decreased by 24.3% in FL and 29.34% in EX, and the stability of M1 and M2 in LB and AR directions was roughly the same. The ROM of M3 decreased further compared with M1, up to 42.01% of FL, and the decrease was smaller in LB and RB. Compared with M2, the ROM of each motion state was reduced by 19.49% ∼ 30.66%. The OS and OP models were similar to the NB group. Compared with N0 and P0, the ROM of N1 and P1 decreased. The ROM of N1 decreased by 42.98% ∼ 78.25% compared with N0 in each motion state, among which LB and RB decreased by 78.25% and 77.26%, respectively, FL and EX decreased by 51.05% and 42.98%, respectively. Similarly, P1 was 72.3% and 70.09% lower than P0 in LB and RB, respectively, and 46.52% and 52.72% lower in FL and EX, respectively. Under the same internal fixation, ROM increased gradually with the decrease of bone mass (Table 3, Figures 3A–C).

**TABLE 3:** L4/5 ROM (°) of the different bone models.

Bone condition	Motion state	INT	M0	M1	M2	M3
NB	FL	4.45	2.76	2.14	1.62	1.24
	EX	3.82	2.31	1.67	1.18	0.95
	LB	4.92	2.26	0.96	1.05	0.93
	RB	5.16	2.34	1.08	1.21	1.05
	LR	2.23	1.24	0.82	0.78	0.56
	RR	2.18	0.96	0.81	0.75	0.52
OS	FL	5.21	3.23	2.55	1.95	1.56
	EX	4.63	3.02	2.64	1.68	1.25
	LB	5.61	2.66	1.22	1.35	1.21
	RB	5.67	2.74	1.29	1.31	1.25
	LR	2.78	1.45	0.86	0.71	0.54
	RR	2.65	1.21	0.82	0.69	0.46
OP	FL	5.89	3.66	3.15	2.36	1.87
	EX	4.97	3.13	2.35	1.79	1.48
	LB	6.21	2.98	1.72	1.86	1.69
	RB	6.32	3.24	1.89	1.91	1.75
	LR	2.96	1.94	1.25	1.08	0.84
	RR	2.85	1.78	1.19	1.12	0.79

### Cortical endplate stress

In all models, the stress of M0 end plate is the largest, and the stress of M3 end plate is the smallest (Table 4). Under NB, compared with M0, M1 reduces the stress of left and right lateral bending and AR. Compared with M1, FE decreased by 67.83%, EX decreased by 60.44%, LB decreased by 35.16%, RB decreased by 34.25%, LR decreased by 71.18%, RR decreased by 69.81%. Compared with M2, the peak stress of M3 upper end plate decreases under various motion states, especially in LR state, with a maximum decrease of 48.42%. The endplate stress of M1 is slightly lower than that of M2, which is reduced by 16.95% and 16.74% respectively. The stress of AR and RR is less than the yield strength of the endplate (60 MPa). The stress of M3 is always less than the yield stress of the endplate under different motion, and <50% under AR and RR. The trend of OS and OP models is the same as that of NB. With the loss of bone mass, the endplate stress of the model gradually increased. Compared with M0, P0 increased the most in LR, which was 46.62%.

**TABLE 4:** Endplate stress (Mpa) for different fixation options of the bone models.

Bone condition	Motion state	M0	M1	M2	M3
NB	FL	145.41	140.01	79.39	45.04
	EX	111.12	124.23	69.25	49.15
	LB	103.26	74.21	89.36	48.12
	RB	102.43	74.68	89.69	49.10
	LR	44.16	37.85	40.67	18.82
	RR	44.08	37.68	40.68	18.81
OS	FL	153.26	124.33	77.98	46.86
	EX	112.85	109.22	67.15	47.01
	LB	111.86	78.81	92.51	47.72
	RB	110.82	79.39	93.00	48.91
	LR	67.49	60.69	41.62	20.46
	RR	67.50	60.56	41.57	20.47
OP	FL	165.83	104.73	75.38	50.37
	EX	111.12	80.52	62.8	46.86
	LB	129.66	86.72	98.16	46.68
	RB	127.99	87.6	99.14	48.45
	LR	82.73	72.72	40.34	20.96
	RR	72.61	69.45	40.30	20.97

### Cage stress

**FIGURE 4:**
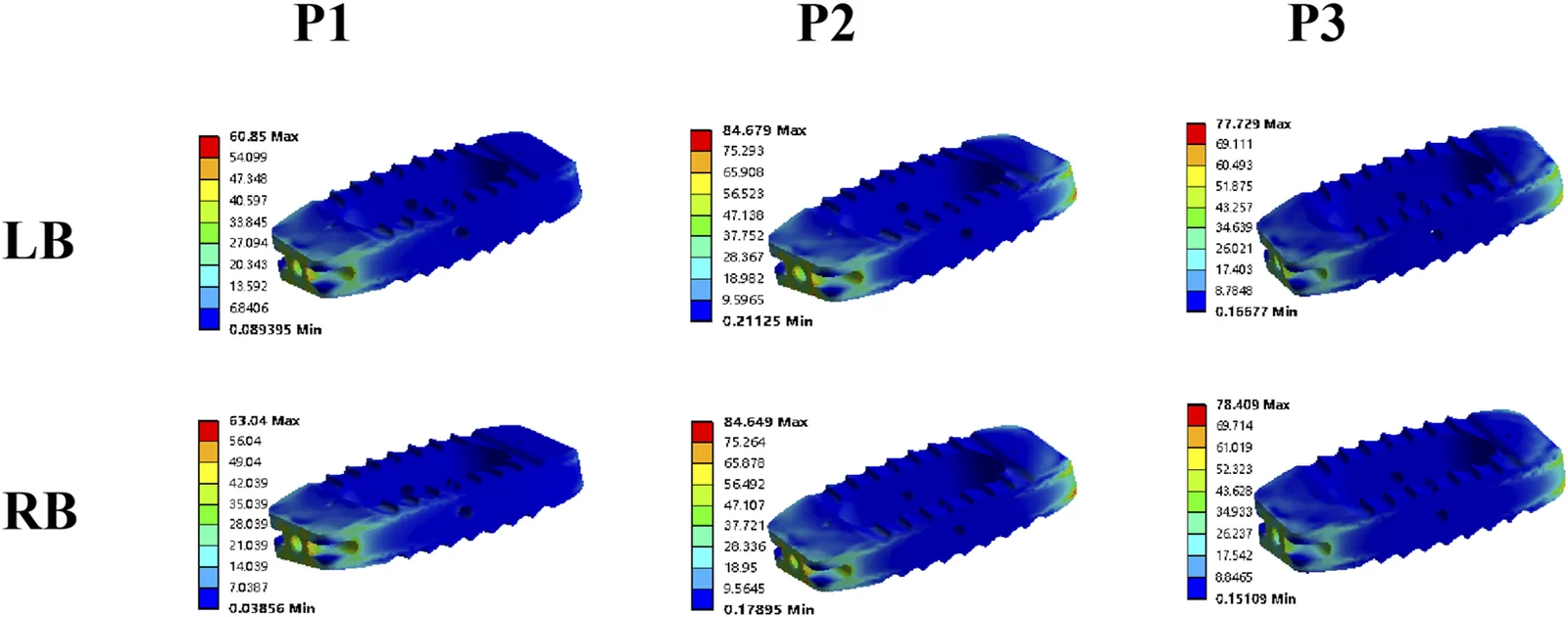
Cage stress (Mpa) of the OLIF Supplemental Fixation in LB and RB directions under OP bone models.

The Cage stress of SA model is the largest, especially FE and EX, and M1 does not change the Cage stress in this motion mode. Except for left and right lateral flexion, the Cage stress of M3 is the smallest in all motion modes. Compared with M0, the Cage stress LB of M1 decreased by 36.73%, and RB decreased by 35.43%. The Cage stress of M2 is less than that of M1 in flexion and extension, higher than that of M1 in lateral bending state, and the stress values are basically similar in rotation. Compared with M1, the peak stress of the Cage of M3 is significantly reduced in both FE and AR motion states. Compared with M2, the peak value of Cage stress of M3 is further reduced, with a decrease of 50.57% at LR and a decrease of 51.16% at RR. The Cage stress trend of OS and OP models is consistent with that of NB. Under the same fixation, the Cage stress of the OP model is greater. Compared with M3, P3 had the largest increase at FE, which was 18.83%. The tensile yield strength of PEEK fusion cage is 96.9 MPa, and the compressive yield strength is 118 MPa (Chen and Lee, 2006). The peak Cage stress of almost all surgical models under each motion is less than the tensile and compressive yield strength (Table 5, Figure 4).

**TABLE 5:** Cage stress (Mpa) for different fixation options of the bone models.

Bone condition	Motion state	M0	M1	M2	M3
NB	FL	96.41	92.46	54.74	39.98
	EX	91.28	87.89	41.97	33.27
	LB	81.15	51.34	71.11	79.99
	RB	80.77	53.49	71.78	80.53
	LR	48.97	45.74	45.56	22.52
	RR	48.99	45.80	45.80	22.52
OS	FL	101.5	95.30	57.31	42.38
	EX	85.40	89.02	42.75	34.35
	LB	85.21	54.34	74.83	84.07
	RB	84.45	56.98	74.30	84.74
	LR	50.55	47.13	46.59	22.71
	RR	50.58	47.20	46.62	22.72
OP	FL	94.37	98.26	62.32	47.51
	EX	89.63	88.07	43.40	35.93
	LB	93.12	60.85	84.68	77.73
	RB	92.39	63.04	84.65	77.41
	LR	54.05	50.17	48.66	22.88
	RR	54.10	50.28	48.71	22.88

### Supplemental fixation stress

**FIGURE 5:**
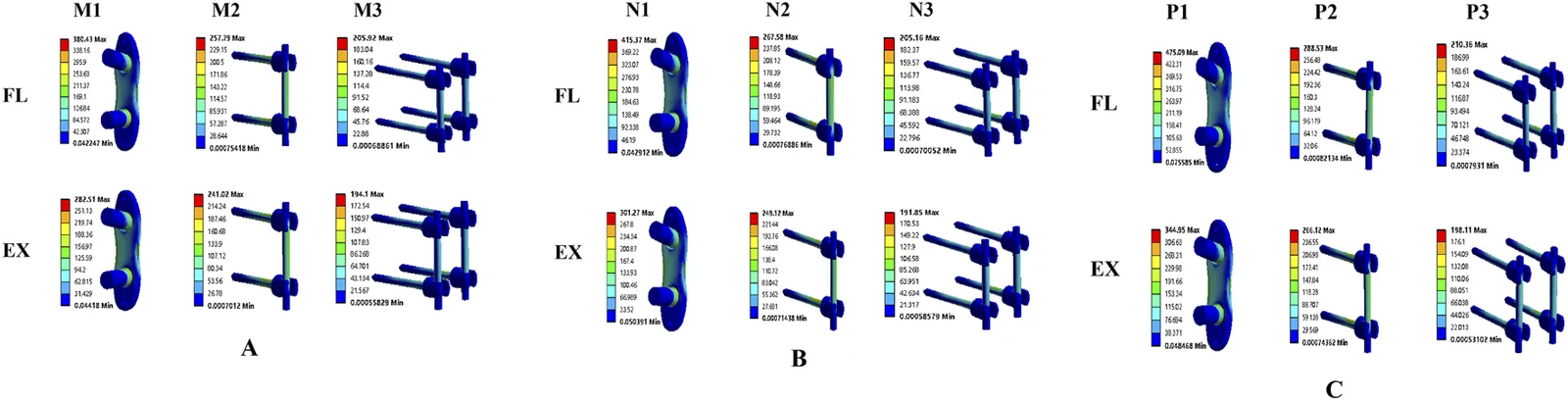
Supplemental Fixation Stress (Mpa) in FL and EX directions under different bones **(A)** NB models. **(B)** OS models. **(C)** OP models.

The stress of M1 internal fixation increased compared with that of M3 in most motion states, which was most obvious in FL and EX, which increased by 40.50% in FL and 31.29% in EX. The internal fixation stress in LB and RB directions is roughly the same. In the NB and OS models, the internal fixation stress of LP under FL exceeded the minimum yield strength of the material by 22.72% and 33.78%, respectively, and the internal fixation stress under other motions was lower than the fatigue strength of 310–610 MPa and the yield strength of 897–1,034 MPa reported in the literature (Fang et al., 2022). In OP, the peak stress of internal fixation exceeded the minimum fatigue strength of the material in FL and EX, and reached 53.25% (>50%) in FL. The peak stress of internal fixation in M2 and M3 models under different motion states is less than the fatigue and yield strength of the material. The peak stress of the M2 model is smaller than that of the M3 model in the LB and RB directions, and the rest is larger than that of the M3 model. Under the same instrument, the peak stress of P3 was significantly higher than that of M3, especially LB and RB, reaching 18.58% and 26.35%, respectively (Figures 5A–C, Table 6).

**TABLE 6:** Fixation stress (Mpa) of the different bone models.

Bone condition	Motion state	M1	M2	M3
NB	FL	380.43	257.79	205.92
	EX	282.51	241.02	194.1
	LB	175.37	146.63	180.04
	RB	175.87	146.09	179.12
	LR	100.6	78.35	60.79
	RR	100.49	78.78	60.82
OS	FL	415.37	267.58	205.16
	EX	301.27	249.12	191.85
	LB	172.86	165.46	199.42
	RB	173.53	164.79	198.29
	LR	100.47	86.66	66.43
	RR	100.35	86.50	66.40
OP	FL	475.09	288.53	210.36
	EX	344.95	266.12	198.11
	LB	167.24	212.38	244.94
	RB	168.36	211.56	243.22
	LR	104.41	109.8	81.91
	RR	104.27	109.56	81.85

## Discussion

The purpose of OLIF arguemented fixation is to create a stable environment for interbody fusion, while effectively sharing the endplate stress and reducing the occurrence of Cage subsidence (Liu et al., 2020). However, due to the decrease of vertebral bone mass and the increase of bone degradation, patients with osteoporosis are prone to Cage subsidence and endplate collapse (Yao et al., 2020). Most of the previous studies have established lumbar fixation models under normal bone, and few studies have studied the effects of bone loss and osteoporosis on interbody fusion. Therefore, we established a three-dimensional, non-linear finite element fixation model with different bone conditions to explore the effect of bone conditions on the combined application of OLIF.

In this study, different internal fixation models were simulated, and the activity of the surgical segment and the stress characteristics of each structure after applying physiological load were verified by the model, and compared with the literature (Dreischarf et al., 2014; Panjabi et al., 1994). The results are basically consistent with previous studies, which proves the effectiveness of the model. The results showed that compared with the intact spine, all fixation methods significantly improved the stability of the lumbar spine structure. M0 decreased by 59.33%, 57.96%, 69.50%, 66.09%, 46.07% and 41.70% in FL, EX, LB, RB, LR and RR movements, respectively, compared with the intact model. This may be related to the larger cage used in OLIF, which increased the contact area between the cage and the endplate, significantly improved the stability of the fixed segment through the interface distraction-compression effect (Song et al., 2021), and reduced the need for additional fixation. This is consistent with the observed clinical phenomenon. However, studies have reported that (Tsai et al., 2016; Chatham et al., 2017), the sinkage of the fuse is closely related to the stress of the end plate and the fuse. In the absence of additional fixation, the weight load is directly distributed on the surface of the fuse and the endplate, increasing the possibility of its sinking (Zhang et al., 2018). In our study, the Cage stress and end plate stress of the SA model under each motion state are greater than those of all internal fixation models, especially the Cage stress is larger in FE and AR. Compared with the non-OP model, the maximum stress of the end plate in the OP model under buckling state increases by 46.62%, which may be a potential risk factor for Cage subsidence. Therefore, for OP patients, auxiliary fixation should be considered to improve the stability of the surgical segment and reduce the risk of internal fixation failure (Zhang et al., 2018).

With the increase of internal fixation, the ROM of the surgical segment showed a downward trend. Compared with M0, the ROM of M1 decreased in all motion directions, and decreased by 51.91%, 56.28%, 80.49%, 79.07%, 63.22% and 62.84% in FL, EX, LB, RB, RB, LR and RR, respectively. In particular, the decrease is most obvious in the left and right flexion directions, which may be related to the fixed position of the LP. LP is fixed on the outside of the vertebral body. For LB and RB movements, the longitudinal axis of the screw is orthogonal to the rotation axis. The reverse pedicle screw trajectory provides a longer lever arm to resist lumbar motion, making the stability significantly improved, slightly higher than M2, and there is no significant difference compared with M3, which also shows the inherent benefits of anterior column fixation. The results of the study are the same as those reported in the literature. Cappuccino A et al. (2010) reported that the LP structure can reduce more than 80% of the lateral motion, which is not significant compared with the BPS structure. Fogel et al. (2014) also reported that the addition of side plates can significantly reduce the ROM of LB, and the stability is comparable to that of BPS structure. These results indicate that LP fixation can effectively control the bending motion, but the stability of LP fixation in the control FL and EX directions is weak. Previous studies have also shown that the lateral plate increases the stiffness of the lumbar spine during LB and AR, but is significantly weaker than posterior pedicle screw fixation during FL and EX (Cappuccino et al., 2010; Zhang et al., 2018). The reason for this result is that LP is fixed on the lateral side, the LP screw intersects the lumbar rotation axis at a smaller angle, and the motion torque is lower than that of the posterior pedicle screw, so the limitation of ROM is very small, while UPS or BPS is fixed on the posterior facet joint. The longitudinal axis of the screw is orthogonal to the lumbar rotation axis, so there is a greater limitation on the ROM in the flexion and extension direction. The results of this study also showed that the L4-5 activity of M2 decreased again on the basis of M1, mainly in the flexion and extension direction, decreased by 24.3% at FL, and decreased by 29.34% at extension. The stability of M1 and M2 in LB and AR directions is roughly the same. The L4-5 activity of M3 was further decreased compared with M1, up to 42.01% of FL, and the decrease was smaller in LB and RB. Compared with M2, the ROM of each motion state decreased by 19.49% ∼ 30.66%, among which RR decreased by 30.66%, EX decreased by 19.49%, which had similar findings in BM model. These results are consistent with previous LLIF biomechanical studies (Fang et al., 2022; Cai et al., 2022; Aoki et al., 2010). BPS further increases the fixed stiffness and reduces the coupling motion effect caused by the unbalanced fixation of LP or UPS (Murphy et al., 2023). Although the ROM of M1 in FE motion state is larger than that of M3 and M4, it is similar to the ROM of M3 under LB motion, and is roughly equivalent to the stability of M2 under AR, indicating that OLIF combined with LP fixation can effectively improve the stability of the fixed segment and promote interbody fusion.

In the OP model, we observed similar results to the NB model and the OS model. However, regardless of the fixation method, the ROM of all movements in the osteoporosis model was larger, the strength of the OP cortical endplate was reduced, the pull-out strength of the internal fixation was weakened, and the ROM limitation was not as good as that of the NB model. When considering the possible complications such as the failure of the internal fixation device after the operation, the strength of the bony structure such as the endplate and the load bearing of the internal fixation should be comprehensively analyzed. Previous studies have shown that LP may increase the stiffness of bending and rotation, but has little effect on flexion and extension (Cappuccino et al., 2010). This is similar to the results of this study. After adding the side plate fixation, the stress of the fusion cage and the end plate decreased to a certain extent in all motion modes, especially in LB and RB modes, which was roughly equivalent to that of BPS fixation. However, the stress of the cage and endplate in LP fixation was greater than that in BPS fixation during FL and EX motion, which may be related to the inherent imbalance of the surgical segment caused by unilateral fixation, resulting in stress redistribution. In this study, we found that the SA model has the highest stress, while the BPS model has the lowest stress. In LP fixation, the inclined trajectory of the screw is subjected to more concentrated stress, resulting in greater internal fixation stress than BPS. Based on our results, in the NB and OS models, the stress of pedicle screw, lateral plate and screws were lower than the fatigue strength of 310 ∼ 610 MPa and yield strength of 897 ∼ 1,034 MPa reported in the literature (Fang et al., 2022), regardless of the way of internal fixation. Therefore, it is feasible and safe to implant lateral plate fixation. For patients with normal bone mass and osteopenia, OLIF combined with LP or UPS fixation can be used as an alternative fixation for BPS. However, we also noted that the stress distribution of LP and BPS was concentrated at the junction of the screw and the vertebral body. Therefore, in order to avoid screw breakage or extraction, we recommend avoiding postoperative overactivity. In OP, the peak stress of the internal fixation exceeds the minimum fatigue strength of the material at FL and EX, and reaches 53.25% (>50%) at FL, which may lead to the loosening or fracture of the internal dislocation device, while the peak stress of the internal fixation in each motion state of the M2 and M3 models is less than the fatigue and yield strength of the material. Therefore, in clinical practice, in the case of OP, it is necessary to consider the limitations of SA, LP, UPS fixation, adjust the auxiliary fixation method of OLIF to adapt to the bone quality of patients, so as to reduce the risk of internal fixation failure, and choose OLIF combined with posterior BPS fixation or more desirable.

### Limitations

Several limitations of this study need to be acknowledged. First, The finite element is based on the geometric information of the human body and cannot perfectly simulate the human body structure. For example, the ligament is simulated as a one-dimensional nonlinear spring element, and the paravertebral soft tissue cannot be accurately reconstructed, ignoring the influence of muscles on the biomechanical function of the spine, the isotropic linear elastic model cannot capture the resistance difference of screw-bone interface in different directions and the directional deviation of local stress concentration. The linear hypothesis ignores the nonlinear behavior of ligament, intervertebral disc and bone tissue under physiological load. Second, The osteoporosis model reduces the elastic modulus of the endplate and bone by a specific ratio, ignoring individual differences; Finally, The model only reflects the immediate biomechanical characteristics after surgery, does not mean long-term postoperative status, and ignores the impact of natural degeneration of the lumbar spine. To a certain extent, the model can only reflect the changes in the biomechanical trend of the lumbar spine under different load conditions, and the results need further clinical verification.

## Conclusion

In summary, the type of internal fixation should be considered according to the actual situation in the clinical application of OLIF. For single-segment degenerative lumbar spine with normal bone mineral density and no obvious sequence imbalance, SA fixation can provide corresponding stability for the surgical segment, while LP fixation and UPS fixation can further improve the stability and reduce the stress of the cage and endplate, so it should be given priority in the application of OLIF. For elderly patients with osteoporosis, it is recommended to combine BPS fixation to maintain postoperative spinal stability and reduce the incidence of postoperative endplate collapse and internal fixation failure.

## Data Availability

The original contributions presented in the study are included in the article/supplementary material, further inquiries can be directed to the corresponding authors.
